# Does Prenatal Physical Activity Affect the Occurrence of Postnatal Anxiety and Depression? Longitudinal Study

**DOI:** 10.3390/ijerph19042284

**Published:** 2022-02-17

**Authors:** Joanna Baran, Katarzyna Kalandyk-Osinko, Rafał Baran

**Affiliations:** 1 Institute of Health Sciences, Medical College, University of Rzeszów, 35-310 Rzeszów, Poland; 2 Natural and Medical Centre for Innovative Research, 35-310 Rzeszów, Poland; 3 Institute of Medical Science, Medical College, University of Rzeszów, 35-310 Rzeszów, Poland; kalandyk@op.pl; 4 Department of Gynaecology and Obstetrics of the Frederic Chopin Clinical Provincial Hospital No. 1 in Rzeszów, 35-055 Rzeszów, Poland; 5 Fortitudo Medical Center, 35-317 Rzeszów, Poland; 6 SOLUTION-Statistical Analysis, 35-120 Rzeszów, Poland; rafal.barann@gmail.com

**Keywords:** anxiety, depression, physical activity, sedentary

## Abstract

The aim of the foregoing study was to assess whether physical activity during pregnancy affects the occurrence of anxiety and depression during pregnancy, postpartum and 6 months following childbirth. This study tried to answer the following questions: How was the incidence of depression and anxiety different in the pre- and postpartum periods? What intensity level of physical activity protects against the symptoms of anxiety and depression? Does the time spent engaged in sedentary activities and MVPA affect the occurrence of depression and anxiety before and after childbirth? The study group under analysis consisted of 187 women aged 19–41 years. The research was conducted between April 2016 and November 2020. The study was divided into four stages: T0—qualification to participate in the study; T1—medical history acquisition, consisting of a short questionnaire and two long questionnaires (the Edinburgh Postnatal Depression Scale (EPDS) and General Anxiety Disorder-7 (GAD-7)), as well as an assessment of 7-day physical activity using Actigraph accelerometers during the pregnancy; T2—the completion of the EPDS and GAD-7 questionnaires after the birth; T3—the completion of the EPDS and GAD-7 questionnaire 6 months after giving birth. The obtained results were statistically processed in the Statistica 13.3 software package. A significance level of *p* < 0.05 was assumed. The highest percentage of depression occurred immediately after the delivery, followed by 6 months after delivery, and the smallest number of women suffered from depression before the birth (*p* < 0.001). The analysis of correlations of physical activity with anxiety symptoms did not show significant correlations. However, the situation is different in the case of depression symptoms. Women taking fewer steps before delivery showed a greater tendency to develop depressive symptoms before, immediately after and 6 months after the delivery (*p* < 0.001). Women who were less active (took fewer steps per day, spent less time in moderate-to-vigorous physical (MVPA) activities or spent more time being sedentary) showed symptoms of depression on the EPDS scale. It appeared that those with severe anxiety symptoms had the highest sedentary time scores before the delivery (*p* = 0.020). Reduced physical activity promotes the onset of postnatal depression, while being active reduces this risk. Interestingly, even light physical activity “protects” against the occurrence of depression and is better than sedentary activities. Such clear conclusions cannot be drawn in relation to anxiety symptoms. Sedentary behaviour may promote anxiety symptoms immediately after childbirth, but this study should be continued in order to confirm it during other time periods.

## 1. Introduction

Physical activity is the healthiest form of spending free time. It has a number of benefits, including reducing body fat, reducing the risk of cancer [1], gallstones [2], gestational diabetes [3] and lowering blood pressure [4]. Some authors believe that exercise is so effective that it should be treated as medicine, but more attention should be paid to the dosage and individual differences between patients [5]. Movement and activity are recommended for everyone, including pregnant women. The prerequisite is an uncomplicated pregnancy.

There are national recommendations for undertaking physical activity during pregnancy, but no worldwide guidelines. The most common absolute contraindications for physical activity include abnormal amniotic fluid, bleeding, cardiovascular diseases, incompetent cervix, diabetes mellitus, foetal abnormality, foetal growth restriction, history of foetal growth restriction, history of miscarriage, ≥3, history of preterm birth, history of preterm labour, infectious disease, lung disease, multiple gestation, placenta previa, preeclampsia, pregnancy-induced hypertension, premature contractions or labour, premature rupture of membranes, uncontrolled thyroid disease, uncontrolled systemic disorder and thrombophlebitis [6].

As mentioned above, physical activity is a very good means to help maintain physical health [7]. It should also be mentioned that it plays a major role in maintaining proper mental condition [8]. According to numerous scientific reports, physical activity supports the functioning of the brain, helps with anxiety [9,10], depression [11,12], addiction [13] and neurodegenerative diseases [14], and supports the mental development of children [15]. Apart from the typically health benefits, it also improves mood and well-being.

Pregnancy, childbirth and the postnatal period are times of enormous changes in a woman’s body. These are physical, hormonal and psychological changes [16,17,18]. One of the common problems that arise during this period is depression (PPD). It can appear before the birth and up to a year after the birth of the child [19]. In the postnatal period, it affects between 6.5 and 20% of women [20,21]. The most common symptoms include sadness, sleep disturbances, lack of interest in the child and a feeling of helplessness [22]. In addition to depression, there can also be anxiety, fear of the new situation after the birth of the child, fear that the mother will not be able to take care of the child or simply fear of giving birth [23,24]. Scientific research confirms that physical activity is a strong preventive measure for cardiovascular diseases and diabetes, but it also has a beneficial effect on the well-being of mothers-to-be, including lowering anxiety and reducing the incidence of postnatal depression [25]. Recent literature reviews indicate that physical activity, both during pregnancy and in the postnatal period, reduces symptoms of postnatal depression [21]. Women who engage in moderate physical exercise in an aquatic environment are less likely to experience PPD than women with a sedentary lifestyle. Overweight and obesity in pregnant women with sedentary lifestyles are closely associated with a positive screening result for PPD [26]. In contrast, the results of a large study, The PAMELA Randomized Clinical Trial, show a different conclusion, with findings that moderate intensity exercise during pregnancy did not lead to a significant reduction in postnatal depression [27]. It is also very important that a woman who has just become a mother and has to devote most of her time to a child, can also do something for herself by spending time actively.

Bearing in mind the above information, the aim of the study was to assess the relationship between physical activity and depression and anxiety in women after childbirth. The following research questions were asked:

1. How was the incidence of depression and anxiety different in the pre- and postpartum periods?

2. What intensity level of physical activity protects against the symptoms of anxiety and depression?

3. Does the time spent engaged in sedentary activities affect the occurrence of depression and anxiety before and after childbirth?

4. Does the time spent engaged in MVPA activity affect the occurrence of depression and anxiety before and after childbirth?

The conducted study is very important both from a scientific and health point of view. Information on whether physical activity reduces the risk of depression and anxiety symptoms, and how intense it needs to be, may be the basis for both prevention and therapy. Sometimes physical activity can protect against pharmacotherapy, which is particularly inadvisable in pregnant women. As a 16th-century royal physician, Wojciech Oczko, used to say: “Movement will replace almost any medicine, while all medicines taken together will never replace movement.”

## 2. Materials and Methods

### 2.1. Participants

In this study, 236 women with uncomplicated pregnancies were invited to participate. Among this group, 215 signed the informed consent form to participate, but 4 women did not obtain the consent of the attending physician to participate in the evaluation (T0). 211 women joined the project (T1). After giving birth, 9 women did not complete the questionnaires and, therefore, did not provide information about their condition (T2), while 6 months after giving birth, 15 women refused to participate in the study or simply did not complete the questionnaire (T3). Finally, the study group submitted for analysis consisted of 187 women aged 19–41 years (average 29.19). 

In addition to the consent of the patient and the attending physician to participate in the study, the condition for participation in the study was meeting the inclusion criteria, which included being over 18 years of age, having an uncomplicated pregnancy and having a singleton pregnancy.

The exclusion criteria included: contraindications to undertake physical activity, lack of consent of the woman for participation in the study at any stage and lack of consent of the attending physician for participating in the study.

In terms of sociodemographic information, the respondents had secondary or higher education, and the vast majority (170) of them were in a formalised relationship. Nearly 60% of the women lived in the city and this was their first to fourth pregnancy. Detailed characteristics of the respondents are provided in Supplementary Tables S1 and S2.

### 2.2. Methods

The research was conducted between April 2016 and November 2020. Women were recruited in the doctor’s surgery. During the follow-up visit, the doctor offered to participate in the study and qualified the patients. If the patient agreed to participate in the study, she made an appointment with a physiotherapist. During this meeting, the physiotherapist interviewed the patient and conducted two questionnaires: the Edinburgh Postnatal Depression Scale (EPDS) and General Anxiety Disorder-7 (GAD-7). Then, they discussed with the patient how to wear the accelerometer and programmed the device. After 7 days of wearing it, the physiotherapist collected the device from the patient. The study was divided into four stages: 

T0—The patient’s consent to participate in the study, and the consent of the attending obstetrician for the patient to participate in the study, was obtained. 

T1—The medical history was acquired, consisting of a short questionnaire and two long questionnaires (EPDS and GAD-7), as well as an assessment of 7 days of physical activity using Actigraph accelerometers during pregnancy. It was worn by all women in the 2nd trimester of pregnancy, i.e., between 20 and 24 weeks of pregnancy.

T2—This stage involved the completion of the EPDS and GAD-7 questionnaires after the birth.

T3—This stage included the completion of the EPDS and GAD-7 questionnaire 6 months after giving birth. 

As mentioned above, in this study, two standardized questionnaires, EPDS and GAD-7, were used. The EPDS questionnaire was used to assess the presence of postnatal depression, as it is the most commonly used screening tool for depression in perinatal care [28,29]. This questionnaire was developed by Cox et al. [30] in 1987. The questionnaire consists of 10 questions rated on a scale from 0 to 3 points. The questions relate to the last 7 days of the respondent’s life. In addition, in order to avoid sequencing error, in some questions the answers are arranged according to scores of 0, 1, 2, 3, and the rest 3, 2, 1, 0. The tool has good psychometric properties. In the original studies, the sensitivity was 86%, the specificity was 78% and the Cronbach’s alpha reliability coefficient was 0.88. The authors of the EPDS scale and the British Journal of Psychiatry, who own the copyrights to the tool, agree to the use and reproduction of the tool provided that the source is cited [30]. The characteristics and psychometric properties of the Polish language version were assessed by Kossakowska [31]. In the literature, many cut-off points for this scale can be found; however, in this study, cut-off points were used according to the most recent study by Levis et al. [32], where depression is detected at 11 or more points.

To assess the occurrence of anxiety states, the GAD-7 questionnaire was used, which is a tool with high criterion accuracy for identifying probable cases of generalized anxiety disorder. The scale is also a very good and reliable measure of symptom severity. The original study conducted in the primary care setting reported that the internal consistency was excellent (Cronbach’s alpha = 0.92) and, at the optimal cut-off value of 10, the sensitivity and specificity were 89.0% and 82.0%, respectively [33]. This is evidenced by the fact that increasing GAD-7 scores are strongly associated with multiple domains of functional impairment and disability. Anxiety levels defined as moderate, moderately severe and severe anxiety in the GAD-7 correspond to cut-off scores of 5, 10 and 15 pts [33]. Numerous studies indicate that GAD-7 represents a clinically useful scale for the detection of GAD in perinatal women [34,35,36]. In Chinese studies, Cronbach’s alpha for the GAD-7 was 0.84, and a sensitivity of 96.8% and a specificity of 56.1% were obtained [37]. In contrast, in the American research on the Spanish-language version of the reliability of the GAD-7, it was evaluated as good (Cronbach’s alpha = 0.89). A cut-off score of 7 or higher, maximizing the Youden Index, yielded a sensitivity of 73.3% and a specificity of 67.3% [36].

Furthermore, an objective measurement tool, the Actigraph wGT3X-BT accelerometer, was used to assess physical activity levels. It is a triaxial device that monitors the subject’s physical activity for a fixed period of time. This device can be safely used by pregnant women, which is confirmed by numerous studies [38,39,40,41]. In the present study, the subjects wore the accelerometer for 7 consecutive days of the week. The accelerometer was put on in the morning after waking up and taken off before going to bed. The device is splash-proof but not waterproof, so the subjects were asked to take it off if they were showering or swimming. Participants were also instructed not to remove the device, e.g., during exercise, while walking or doing housework.

Using the Actigraph, the following parameters were assessed in the physical activity assessment: sedentary (time spent in sedentary activity in min.), light (time spent in light activity in min.), moderate (time spent in moderate activity in min.), vigorous (time spent in vigorous activity in min.), % in sedentary (% time spent in sedentary activity), % in light, (% time spent in light activity), % in moderate (% time spent in moderate activity), % in vigorous (% time spent in vigorous activity), total moderate-to-vigorous physical activity (MVPA) (total time spent in moderate-to-vigorous activity in min), % in MVPA (% time spent in moderate to vigorous activity), average MVPA per day (average time spent in moderate to vigorous activity per day in min.), step counts and step counts per day.

The result was considered as a valid one if the subject wore the accelerometer for at least 4 days a week (for a minimum of 500 min a day), of which there were at least 3 weekdays and 1 day out of the weekend days [42].

Such a selection of research tools resulted primarily from their measurement reliability and credibility, as well as their safety and the possibility of using them in pregnant women. While the EPDS is a scale dedicated to pregnant women, the GAD-7 scale and accelerometers are general purpose, but have been tested for use in pregnant women. The quoted references confirm the usefulness of the above-mentioned scales and accelerometers in conducting research on pregnant women.

### 2.3. Statistical Analysis

The obtained results were statistically processed in the Statistica 13.3 software package. Descriptive statistics were determined (N, $\bar{x}$, Me, Min, Max, Q1, Q3, SD, 95% CI) and the normality of the distribution of variables was checked using the Shapiro–Wilk test. The Friedman ANOVA test was used to assess differences in the prevalence of depression and anxiety in the 3 stages of the study. Spearman’s R correlations between physical activity parameters and scores on the EPDS and GAD-7 scales were also determined. Student’s *t*-test for independent samples, the Mann–Whitney U test and the Kruskal–Wallis test were used to calculate the relationship between physical activity and the occurrence of depression and anxiety. A significance level of *p* < 0.05 was assumed.

## 3. Results

First, the frequency of depression and anxiety symptoms in the study group was determined. The prevalence of postnatal depression and anxiety related to the parturition was analysed before the birth, after the parturition and 6 months following childbirth. The highest percentage of depression occurred immediately after the delivery, followed by 6 months after delivery, and the smallest number of women suffered from depression before the birth (*p* < 0.001).

Moderate and severe anxiety symptoms were most common in women before the delivery and decreased with time (*p* < 0.001). Detailed data are presented in Table 1.

Then, the analysis of correlations of physical activity with anxiety symptoms was performed, and this did not show significant correlations. However, the situation is different in the case of depression symptoms. There was a high and very high correlation with the number of steps taken. Women taking fewer steps before delivery showed a greater tendency to experience depressive symptoms before, after and 6 months after the delivery (*p* < 0.001). The strongest relationship occurred 6 months after the delivery. Furthermore, any physical activity undertaken prior to the delivery resulted in lower scores on the EPDS scale, reflected as statistically significant correlations with varying levels of power. In addition, we also found that the more time women spent in sedentary activities before the delivery, the higher their EPDS scale scores after the delivery were (*p* = 0.008). Detailed data are shown in Table 2.

The next stage of the study was to determine whether the physical activity of women with or without symptoms of depression differs in the three stages assessed. It has been shown that, in all cases, the differences were found to be statistically significant (range of statistical significance from *p* < 0.001 to *p* = 0.007). Women who were less active (took fewer steps per day, spent less time in MVPA activities or spent more time being sedentary) showed symptoms of depression on the EPDS scale (Table 3).

For anxiety, there was only one statistically significant relationship between average daily time in sedentary activities and postnatal anxiety symptoms. It appeared that those with severe anxiety symptoms had the highest sedentary time scores before the delivery (*p* = 0.020, Table 4).

## 4. Discussion

Physical activity has been an extremely popular subject in recent years. There is a growing awareness of the positive role and the benefits of physical activity [43,44]. Its main advantage is that it can be performed anywhere, in a sports club, in the comfort of one’s own home and in an organised or individual form, following the principle that any physical activity is better than no activity at all.

Pregnant women are also more and more willing to engage in physical activity for a variety of reasons. This may be a desire to look after their health, to have a nice figure and to become fit more quickly after giving birth, or as preparation for childbirth. There are cases when women are afraid that exercises may be harmful to the baby; however, this happens less and less frequently.

Unfortunately, pregnancy, childbirth and the postnatal period are increasingly becoming a difficult time for women, which can lead to depression and anxiety. This study was conducted to determine whether being active during pregnancy can protect women from these problems. It turned out that the most difficult period for the participants was immediately after giving birth. This is when the highest percentage of depression symptoms was recorded. The world literature reports very divergent results regarding the incidence of PPD, with rates ranging from 7 to 20% [45] up to 60.8% [46].

The analysis of our results clearly showed that exercise during pregnancy provides better mental well-being after the delivery. Women who were physically inactive or with reduced activity were more likely to suffer from depression symptoms than active women. Furthermore, women with depression were nearly half as active as women without depression. This is consistent with the results of other researchers [47,48].

It is also very important to note that even light physical activity protected women from depression symptoms (as time in activity increased, women scored fewer points on the EPDS scale). 

Sometimes, more activity does not mean better protection. Susukida’s study found that, most often, those women who performed only light physical activity during pregnancy were significantly less likely to experience psychological stress during pregnancy, while those women who performed a combination of light, moderate and vigorous physical activity during pregnancy were more likely to experience psychological distress, stress during pregnancy and depression after giving birth [49]. The study by Demissie et al. showed that, although previous work has indicated that recreational physical activity may be a useful tool for preventing and controlling depression in the postnatal period, their study does not support these findings. Their study found no benefit in the likelihood of depression symptoms from recreational MVPA and found that active postpartum women may have an increased risk of depression, especially those who participate in adult- and childcare and MVPA in the household [50]. These results are different from our own study; however, it should be taken into account that environmental influences were also analysed, which we did not consider in our study.

Interestingly, the analysis in terms of the association between physical activity and anxiety symptoms showed that postpartum women with severe anxiety symptoms had the longest duration of sedentary activity during pregnancy. This is an interesting observation that is worth developing in future studies when considering the influence of other factors, e.g., sociodemographic or economic factors. 

The evidence found in the literature suggests that the combined risk of poor mental health and low levels of physical activity make women vulnerable to complications in pregnancy [51]. In contrast, supervised physical activity during pregnancy may be a good approach to preventing and reducing symptoms of anxiety and prenatal anxiety [52]. Regular physical exercise during pregnancy may be a factor in the prevention of mental health disorders in pregnant women [53]. 

To sum up, the conducted study, despite the authors’ efforts, has some limitations. One of them is the assessment of physical activity only in the prenatal period. The second one is the lack of taking into account the sociodemographic and socioeconomic factors when assessing interactions between depressive and anxiety symptoms and the physical activity level. The strengths of the study include the objective assessment of physical activity with a reliable post-measurement tool, i.e., the Actigraph accelerometer, as well as the fact that the examined patients were during the same period of pregnancy. The study was performed at three time intervals with a relatively large number of respondents, taking into account the scope of the study. All these measurements were made in direct contact with the patient. 

## 5. Conclusions

The postpartum period is characterized by a higher incidence of depression than during pregnancy. On the other hand, during pregnancy, women are more likely to experience moderate to severe anxiety symptoms. This could be because of the fear of childbirth, a new situation, or a fear for the health of the baby. The conducted studies have shown a high correlation between physical activity during pregnancy and mental health before and after delivery. Reduced physical activity promotes the onset of postnatal depression, while being active reduces this risk. Interestingly, even light physical activity “protects” against the occurrence of depression and is better than sedentary activities. Additionally, the time spent in MVPA activity strongly differentiates the occurrence of depression.

Such clear conclusions cannot be drawn in relation to anxiety symptoms. Sedentary behaviour may promote anxiety symptoms immediately after childbirth, but this study should be continued in order to confirm it during other time periods.

In conclusion, the results obtained encourage the continuation of the topic, extending it with new aspects. Making some modifications to the research methodology will allow us to reduce the limitations of the conducted research and to improve the analyses. Considering environmental factors and physical activity in the occurrence of symptoms of depression or anxiety would make it possible to perform a regression analysis indicating which of the factors potentially influences the occurrence of mental disorders the most. By carrying out an assessment of physical activity after childbirth, it would be possible to compare how it changes with the arrival of the child in the world. Finally, carrying out an activity analysis in all three trimesters of pregnancy would make it possible to check which period of activity is most important to prevent a woman from developing depression or anxiety.

## Figures and Tables

**Table 1:** The occurrence of depression and anxiety in pregnant women and after childbirth.

	Before the Birth		After the Parturition		6 Months Following Childbirth		Significance (*p*)
	N	%	N	%	N	%	
EPDS							
0–10	177	94.7	145	77.5	157	84.0	*p* < 0.001 *
>11	10	5.3	42	22.5	30	16.0	
Total	187	100.0	187	100.0	187	100.0	
GAD-7							
Mild (0–4 pkt)	60	32.1	65	34.8	112	59.9	*p* < 0.001 *
Moderate (5–9 pkt)	104	55.6	94	50.3	61	32.6	
Moderately severe (10–14 pkt)	15	8.0	23	12.3	14	7.5	
Severe (15–21 pkt)	8	4.3	5	2.7	0	0.0	
Total	187	100.0	187	100.0	187	100.0	

* Friedman ANOVA.

**Table 2:** Analysis of the correlation of physical activity with the occurrence of anxiety and depression symptoms.

Variables	GAD-7			EPDS		
	Before the Birth	After the Parturition	6 Months Following Childbirth	Before the Birth	After the Parturition	6 Months Following Childbirth
Step counts per day	0.00 (*p* = 0.927)	0.01 (*p* = 0.916)	0.10 (*p* = 0.174)	−0.63 (*p* < 0.001)	−0.59 (*p* < 0.001)	−0.73 (*p* < 0.001)
Average MVPA per day (min)	0.01 (*p* = 0.925)	0.02 (*p* = 0.746)	0.11 (*p* = 0.126)	−0.43 (*p* < 0.001)	−0.38 (*p* < 0.001)	−0.49 (*p* < 0.001)
Total MVPA (min)	0.02 (*p* = 0.797)	0.05 (*p* = 0.494)	0.11 (*p* = 0.145)	−0.41 (*p* < 0.001)	−0.34 (*p* < 0.001)	−0.44 (*p* < 0.001)
% in MVPA	−0.09 (*p* = 0.235)	−0.06 (*p* = 0.413)	−0.01 (*p* = 0.902)	−0.36 (*p* < 0.001)	−0.34 (*p* < 0.001)	−0.42 (*p* < 0.001)
Sedentary (min)	0.03 (*p* = 0.713)	0.09 (*p* = 0.199)	0.05 (*p* = 0539)	0.09 (*p* = 0.196)	0.19 (*p* = 0.008)	0.11 (*p* = 0.131)
Light (min)	−0.04 (*p* = 0.551)	0.03 (*p* = 0.645)	0.06 (*p* = 0.382)	−0.42 (*p* < 0.001)	−0.30 (*p* < 0.001)	−0.40 (*p* < 0.001)
Moderate (min)	0.01 (*p* = 0.927)	0.01 (*p* = 0.852)	0.09 (*p* = 0.209)	−0.36 (*p* < 0.001)	−0.30 (*p* < 0.001)	−0.41 (*p* < 0.001)
Vigorous (min)	0.02 (*p* = 0.789)	0.15 (*p* = 0.043)	0.09 (*p* = 0.245)	−0.23 (*p* = 0.002)	−0.20 (*p* = 0.006)	−0.21 (*p* = 0.005)
% in Sedentary	0.03 (*p* = 0.715)	0.02 (*p* = 0.831)	−0.04 (*p* = 0.617)	0.33 (*p* < 0.001)	0.33 (*p* < 0.001)	0.35 (*p* < 0.001)
% in Light	−0.08 (*p* = 0.305)	−0.09 (*p* = 0.199)	−0.06 (*p* = 0.400)	−0.22 (*p* = 0.002)	−0.24 (*p* = 0.001)	−0.21 (*p* = 0.004)
% in Moderate	0.02 (*p* = 0.768)	0.00 (*p* = 0.966)	0.08 (*p* = 0.299)	−0.33 (*p* < 0.001)	−0.34 (*p* < 0.001)	−0.40 (*p* < 0.001)
% in Vigorous	0.00 (*p* = 0.985)	0.10 (*p* = 0.177)	0.04 (*p* = 0.592)	−0.15 (*p* = 0.039)	−0.16 (*p* = 0.027)	−0.13 (*p* = 0.081)

Spearman’s R correlation.

**Table 3:** The relationship between physical activity and the occurrence of depression.

EPDS	Before the Birth		After the Parturition		6 Months Following Childbirth	
	With Depression >11	Without Depression 0–10	With Depression >11	Without Depression 0–10	With Depression >11	Without Depression 0–10
Step counts per day	4257.27	8699.03	5710.36	9258.39	5159.04	9092.55
Significance (*p*)	*p* < 0.001 **		*p* < 0.001 *		*p* < 0.001 **	
Average MVPA per day	48.93	117.54	72.18	125.94	59.99	124.16
Significance (*p*)	*p* = 0.003 **		*p* < 0.001 **		*p* < 0.001 **	
Average sedentary activity per day	980.84	588.50	752.76	567.98	792.92	574.43
Significance (*p*)	*p* = 0.005 **		*p* = 0.007 **		*p* = 0.002 **	

* Student’s *t*-test for independent samples; ** Mann–Whitney U Test.

**Table 4:** The relationship between physical activity and the occurrence of anxiety symptoms.

GAD−7	Before the Birth				After the Parturition				6 Months Following Childbirth			
	Mild (0–4)	Moderate (5–9)	Moderately Severe (10–14)	Severe (15–21)	Mild (0–4)	Moderate (5–9)	Moderately Severe (10–14)	Severe (15–21)	Mild (0–4)	Moderate (5–9)	Moderately Severe (10–14)	Severe (15–21)
Step counts per day	8592.3	8452.9	8009.9	8438.8	8221.2	8567.0	9471.9	7148.92	8305.5	8792.9	8265.5	-
Significance (*p*)	*p* = 0.846 *				*p* = 0.250 *				*p* = 0.206 *			
Average MVPA per day	114.3	111.4	113.2	144.0	116.6	104.8	131.1	101.6	107.8	119.4	138.5	-
Significance (*p*)	*p* = 0.932 *				*p* = 0.558 *				*p* = 0.524 *			
Average sedentary activity per day	574.9	627.8	657.0	542.2	659.5	562.9	483.6	854.5	597.3	627.8	627.2	-
Significance (*p*)	*p* = 0.887 *				*p* = 0.020 *				*p* = 0.946 *			

* Kruskal–Wallis Test.

## Data Availability

The data presented in this study are available on request from the corresponding author.

## Supplementary Materials

The following supporting information can be downloaded at: https://www.mdpi.com/article/10.3390/ijerph19042284/s1, Table S1: Characteristics of the study group; Table S2: Sociodemographic characteristics of the study group.
