# Supplementary material for: Does Prenatal Physical Activity Affect the Occurrence of Postnatal Anxiety and Depression? Longitudinal Study
Source: Int J Environ Res Public Health. 2022 Feb 17;19(4):2284. doi: 10.3390/ijerph19042284 (PMC8872187; doi:10.3390/ijerph19042284)
Supplement: Supplementary file 1 [file ijerph-19-02284-s001.zip › ijerph-1528920-supplementary.pdf]

Supplementary Table S1. Characteristics of the study group.

Variables	Descriptive statistics									
	N	$\overline{x}$	Me	Min.	Max.	Q1	Q3	SD	CI −95.0%	CI +95.0%
**Age [years]**	187	29.19	29.00	19.00	41.00	26.00	31.00	4.01	3.64	4.47
**Body height [cm]**	187	165.05	165.00	150.00	187.00	160.00	170.00	6.19	5.62	6.89
**Body mass [kg]**	187	66.93	66.00	45.00	112.00	60.00	73.00	10.53	9.56	11.72
**BMI**	187	24.50	24.17	17.91	36.81	22.15	26.04	3.10	2.81	3.45
**Number of pregnancy**	187	1.77	2.00	1.00	4.00	1.00	2.00	0.80	0.73	0.89
**Sedentary [min]**	187	3953.19	3240.42	510.25	9911.8	2544.92	4383.67	2170.49	1970.55	2415.94
**Light [min]**	187	1090.08	1117.42	70.83	2080.50	846.17	1334.67	363.40	329.92	404.49
**Moderate [min]**	186	626.62	355.13	48.08	2105.00	199.92	1061.67	499.18	453.08	555.80
**Vigorous [min]**	187	102.48	76.50	0.00	516.08	0.00	158.33	114.66	104.10	127.63
**% in Sedentary**	187	66.07	67.00	31.00	99.00	57.43	74.22	13.10	11.89	14.58
**% in Light**	187	20.89	22.10	1.00	36.00	17.33	26.00	7.17	6.51	7.98
**% in Moderate**	187	11.15	6.38	1.00	39.00	4.25	19.00	8.67	7.87	9.65
**% in Vigorous**	187	1.85	1.58	0.00	7.00	0.00	3.15	1.88	1.71	2.10
**Total MVPA [min]**	187	730.80	538.42	64.42	2156.17	321.50	1071.08	511.67	464.54	569.53
**% in MVPA**	187	12.18	9.51	0.22	38.88	5.99	18.46	7.95	7.22	8.85
**Average MVPA per day [min]**	187	113.87	82.15	9.20	308.02	53.58	162.17	73.53	66.76	81.84
**Steps Counts**	187	53475.50	54253.00	3320.00	105283.00	38932.00	65488.00	19470.30	17676.74	21672.11
**Steps counts per day**	187	8461.50	8774.00	474.29	15040.43	6791.00	10053.50	2658.75	2413.83	2959.42
**GAD7 before birth**	187	6.19	6.00	1.00	18.00	4.00	7.00	3.33	3.02	3.70
**GAD7 after childbirth**	187	6.11	6.00	1.00	16.00	4.00	8.00	3.31	3.00	3.68
**GAD7 6 months after childbirth**	187	4.37	4.00	1.00	13.00	2.00	6.00	2.71	2.46	3.02
**EDSP before birth**	187	5.13	5.00	1.00	13.00	3.00	7.00	2.81	2.55	3.12
**EDSP after childbirth**	187	7.98	7.00	3.00	19.00	5.00	10.00	3.51	3.19	3.91
**EDSP 6 months after childbirth**	187	5.79	5.00	1.00	18.00	3.00	7.00	3.84	3.49	4.27

Supplementary Table S2. Sociodemographic characteristics of the study group.

	N	N All	%	Procent All
**Place of residence**				
**Urban area**	109	109	58.29	58.3
**Rural area**	78	187	41.71	100.0
**Education**				
**Basic**	0	0	0.00	0.0
**Professional**	0	0	0.00	0.0
**Medium**	46	46	24.60	24.6
**Higher**	141	187	75.40	100.0
**Marital status**				
**Single**	17	17	9.09	9.1
**In a relationship / married woman**	170	187	90.91	100.0
**Income**				
**to 1000zł**	9	9	4.81	4.8
**1000–1500 PLN**	15	24	8.02	12.8
**1500–2000 PLN**	54	78	28.88	41.7
**2000–2500 PLN**	68	146	36.36	78.1
**2500–3000 PLN**	31	177	16.58	94.7
**3000–4000 PLN**	8	185	4.28	98.9
**above 4000 PLN**	2	187	1.07	100.0
